# The expression of ACAT1 in oral squamous cell carcinoma and the adjacent pre-tumour tissue

**DOI:** 10.37796/2211-8039.1363

**Published:** 2022-12-01

**Authors:** Hadis Firouzpour, Seyed Mohammad Shokrolahi, Ekaterina Bourova-Flin, Samira Derakhshan, Zahra Shahsavari, Abbas Karimi, Hassan Mir Mohammad Sadeghi, Afsaneh Goudarzi

**Affiliations:** a Department of Clinical Biochemistry, School of Medicine, Shahid Beheshti University of Medical Sciences, Tehran, Iran; b CNRS UMR 5309/INSERM U1209/University Grenoble-Alpes/Institute for Advanced Biosciences, La Tronche, France; c Oral and Maxillofacial Pathology Department, School of Dentistry, Tehran University of Medical Sciences, Tehran, Iran; d Department of Oral and Maxillofacial Surgery, School of Dentistry, Tehran University of Medical Sciences, Tehran, Iran; e Department of Oral and Maxillofacial Surgery, School of Dentistry, Shahid Beheshti University of Medical Sciences, Tehran, Iran

**Keywords:** ACAT1, OSCC, Metabolism

## Abstract

**Background:**

Altered acetyl CoA acetyltransferase 1 (ACAT1) expression has been reported in diverse cancers. However, the expression of ACAT1 and its prognostic value in oral squamous cell carcinoma (OSCC) has remained unexplored.

**Materials and methods:**

In this study, the expression of ACAT1 was analysed by immunohistochemistry (IHC) in 61 OSCC patients and compared between OSCC and adjacent pre-tumour tissue of 21 patients.

**Results:**

The expression of ACAT1 in OSCC tumours is heterogeneous between patients. More specifically, 52.38% of the patients show low expression of ACAT1 in both tumour and adjacent pre-tumour tissues, 9.52% of the patients show high expression of ACAT1 in both tumour and adjacent pre-tumour, 19.05% of the patients have high expression of ACAT1 in tumour tissue and low expression of ACAT1 in adjacent pre-tumour tissue and another 19.05% of the patients have low expression of ACAT1 in tumour tissue and high expression of ACAT1 in adjacent pre-tumour tissue.

**Conclusion:**

Comparison of ACAT1 expression, one of the key enzymes in the ketone body metabolic pathway, divided OSCC patients into two groups: 1) similar expression and 2) different expression of ACAT1 in tumour and adjacent pre-tumour tissue. No significant association between ACAT1 levels and overall survival was observed.

## 1. Introduction

Oral squamous cell carcinoma (OSCC) is the most common malignancy of oral cancer with high mortality and 5-year survival is less than 50% [1]. There is increasing evidence supporting the link between altered metabolism and cancer [2,3]. The metabolic dysregulation favours tumour growth and identification of changes in gene expression of enzymes involved in metabolic pathways could expand the panel of cancer biomarkers as well as therapeutic strategy [2,3]. Mitochondrial acetyl-CoA acetyl transferase 1 (ACAT1) was previously recognized as a thiolase enzyme involved in the ketone body metabolism, fatty acid beta oxidation and isoleucine degradation pathway [4]. Interestingly, several studies in diverse cancers revealed the oncogenic function of ACAT1 recently, which occurs through several mechanisms [5,6]. ACAT1 has been discovered to have acetyltransferase activity, which can acetylate several metabolic enzymes including pyruvate dehydrogenase alpha (PDHA1) and pyruvate dehydrogenase phosphatase1 (PDP1) leading to inhibition of PDHA1 and PDP1 and Warburg effect [6]. In addition, a recent study by Fan et al. showed that active tetrameric ACAT1 promotes tumour growth while its disruption to monomeric form attenuates cancer cell proliferation [6]. The altered expression level of ACAT1 has been reported in clear renal cell carcinoma, prostate cancer and glioblastoma [5,7,8] and the understanding of the molecular mechanisms underlying its oncogenic role would still require further research. Additionally, since cancer cells require a high level of energy to fulfill their rapid proliferation, the high level of ketone body metabolic enzymes could help cancer cells provide energy from both carbohydrates and ketone bodies, therefore in cancer with a low level of ketone body metabolic enzymes, carbohydrate restriction and ketogenic diet (KD) therapy could be useful to inhibit tumour growth [7,9–14]. This study aimed to investigate the expression level of ACAT1 between OSCC and the adjacent pre-tumour tissues and its association with the survival of OSCC patients. Our study highlights differences in protein expression levels of ACAT1 between OSCC patients, as well as between OSCC and adjacent pre-tumour tissues. These results suggest that only a few OSCC cases with low expression of ACAT1 may respond to KD therapy, although additional investigations of other enzymes related to the ketolysis would be required.

## 2. Methodology

### 2.1. Patients

Formalin-fixed paraffin-embedded fresh human OSCC and adjacent pre-tumour biopsy specimens were selected to evaluate the expression of ACAT1 between OSCC and adjacent pre-tumour tissues. These included 21 OSCC samples with their adjacent pre-tumour tissues that were harvested at surgical removal from hospitals (Bahman and Shariati, Tehran, Iran). All the OSCC patients were histopathologically diagnosed. Fresh tissues were immediately fixed in formalin for histological study. Adjacent pre-tumour tissues were harvested far from the margin of tumours. Additionally, 40 OSCC paraffin embedded tissues without adjacent pre-tumour tissues were obtained from the archives of the Oral and Maxillofacial Department, Tehran Medical University. Altogether, an overall of 61 OSCC patients with paraffin blocks were used to evaluate the association of ACAT1 expression with survival probabilities. This research project was approved by the Ethics Committee of Shahid Beheshti University of Medical Sciences (IR.SBMU.MSP.REC.1399.528).

### 2.2. Immunohistochemistry

The formalin-fixed tissues obtained at surgical removal were dehydrated and embedded in paraffin. Four-micrometer-thick paraffin sections were prepared from OSCC and adjacent pre-tumour tissues, followed by deparaffinization in xylene and rehydration in serial dilutions of ethanol. The tissues were incubated in PBS 3% hydrogen peroxide for 10 min. Next, the antigen retrieval was performed using citrate buffer (pH = 6) at 90 °C for 15 min. The tissues were then incubated with the ACAT1 primary antibody (Sigma HPA007569, 1/200) for 1 h at RT, followed by three washes with PBS and incubation with primary antibody amplifier master for 20 min, after washing with PBS, slides were incubated with the secondary antibody for 30 min, followed by washing in PBS. The signal was developed with DAB substrate solution, then counterstained with hematoxylin and dehydrated with serial dilutions of alcohol and mounted.

### 2.3. Evaluation of immunohistochemical staining

**Fig. 1:**
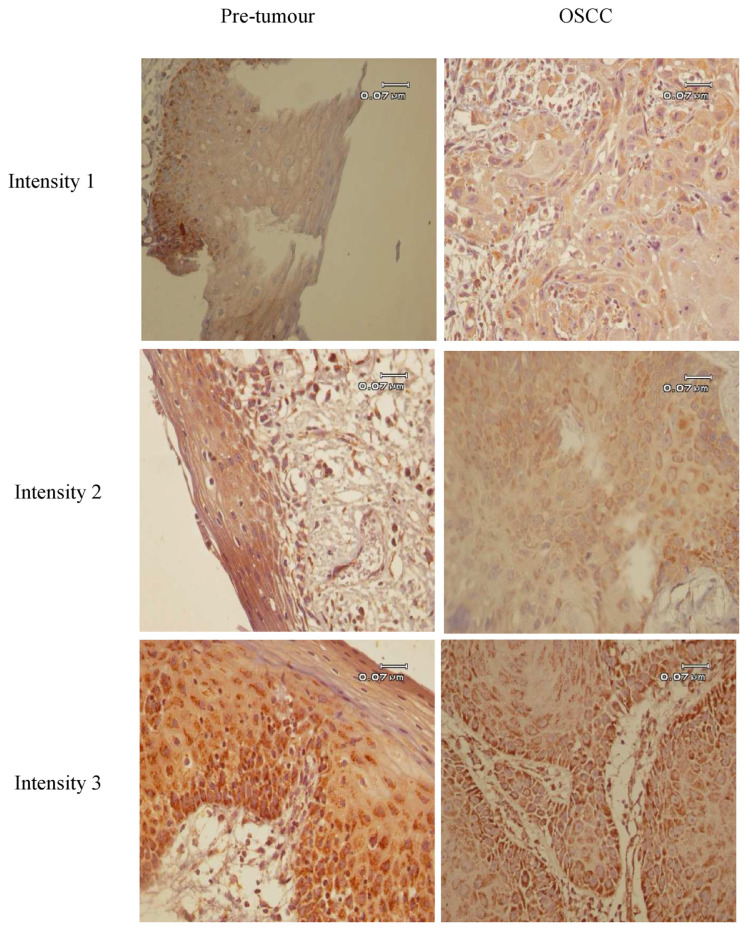
Images of OSCC tissue samples immunostained with ACAT1 antibody showing the intensity scores from 0 to 3. IHC assay was performed on paraffin-embedded sections of OSCC tissues using anti-ACAT1. The scores of signal intensities were assigned to ACAT1 as: negative (0), weak [1], moderate [2] and strong [3] using a light microscope. Original magnification, 400×; Scale bar = 0.07 μm.

The stained slides were observed using a light microscope. For each slide, the intensity staining score was applied as follows: 0 (negative), 1 (weak), 2 (moderate) and 3 (strong)) (Fig. 1). The score of positively stained cells were defined as follows: 0 (negative), 1 (1–25%), 2 (26–50%), 3 (51–75%) and 4 (76–100%). The total staining score of ACAT1 was calculated in five randomly selected fields according to the multiplication of the intensity score to the score of positively stained cells. The high and low expression of ACAT1 were considered based on the total staining score of ACAT1 ≥4 and < 4, respectively.

### 2.4. Statistical analysis

The statistical analysis was performed using Graphpad Prism software version 8. The ACAT1 percentage of positive cells and its total score were compared between OSCC and adjacent pre-tumour tissues using paired sample T test. Survival analysis was performed using the Kaplan–Meier method and log-rank test with Python software and the lifelines package. p-values < 0.05 were considered statistically significant.

## 3. Results

### 3.1. ACAT1 protein expression in OSCC and adjacent pre-tumour tissues

**Fig. 2:**
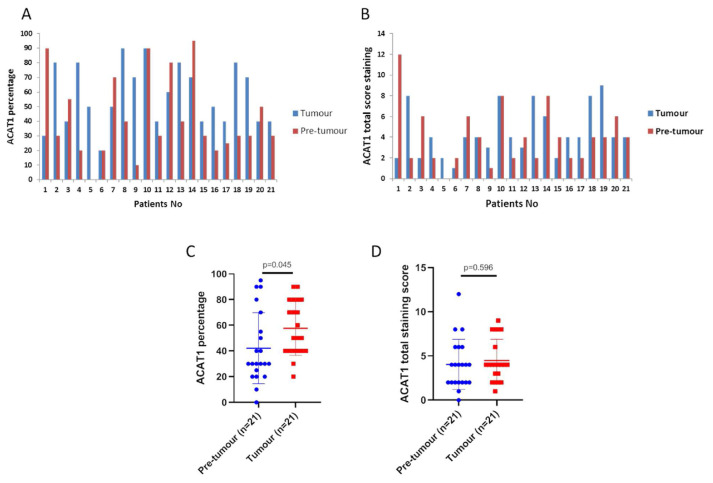
Comparison of ACAT1 scores between OSCC and adjacent pre-tumour tissues. (A) The percentage score of tumour and adjacent pre-tumour tissues (B) Similarly, the ACAT1 total score of the tumour and adjacent pre-tumour tissues. (C and D) The results of paired sample t-test comparing percentage and total score of ACAT1 between OSCC and adjacent pre-tumour tissues: *p-value <0.05.

**Fig. 3:**
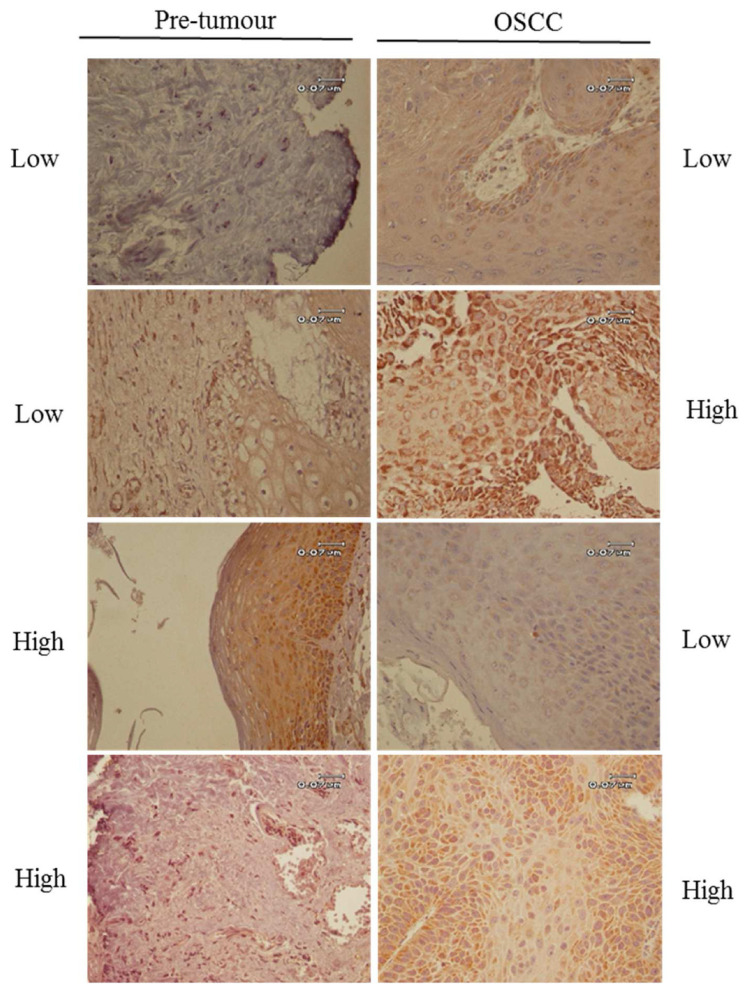
Differential expression of ACAT1 in OSCC and adjacent pre-tumour tissues. Representative IHC results of 4 OSCC cases illustrate low and high ACAT1 expression in OSCC and adjacent pre-tumour tissues. The four observed pattern of ACAT1 expression including OSCC^low^/Normal^low^, OSCC^high^/Normal^low^, OSCC^low^/Normal^high^ and OSCC^high^/Normal^high^ are shown from top to the bottom of panel. Scale bar: 0.07 μm.

The results from IHC performed on 61 OSCC tissues using ACAT1 antibody showed that ACAT1 is variably expressed among OSCC tumours, with high ACAT1 protein expression in 23 of 61 patients (37.7%) and low ACAT1 protein expression in 38 of 61 patients (62.3%). We then evaluated the ACAT1 expression in adjacent pre-tumour tissues and looked for possible differences with OSCC tumour tissue. To this end, we took advantage of 21 samples for which both OSCC tumour and adjacent pre-tumour tissues were available. Variable ACAT1 protein expression was observed in pre-tumour tissues, similar to OSCC tissues. Indeed, out of the 21 patients, high ACAT1 protein expression was found in the pre-tumour tissue of 6 patients (29%) and low ACAT1 protein expression in 15 patients (71%). We then wanted to evaluate whether the percentages of ACAT1 positively stained cells and ACAT1 total score staining were similar or different between tumour and their adjacent pre-tumour matched tissues in our 21 patients. We performed a paired sample T test and found a higher percentage of ACAT1 positive cells in tumour tissues compared to adjacent normal mucosa tissues (p-value = 0.045) whereas no significant difference was observed between the total scores of tumour and adjacent pre-tumour tissues (p-value = 0.596) (Fig. 2). Additionally, the comparison of ACAT1 protein expression according to the high and low level of ACAT1 total score staining identified four groups of patients, which include i/OSCC^low^/Normal^low^ (11 out of 21 patients (52.38%)), ii/OSCC^low^/Normal^high^ (4 of 21 patients (19.05%)), and iii/OSCC^high^/Normal^low^ (4 of 21 patients (19.05%)) and iv/OSCC^high^/Normal^high^ (2 of 21 patients (9.52%)) (Fig. 3) (Table S1).

### 3.2. ACAT1 protein expression and patient survival

**Fig. 4:**
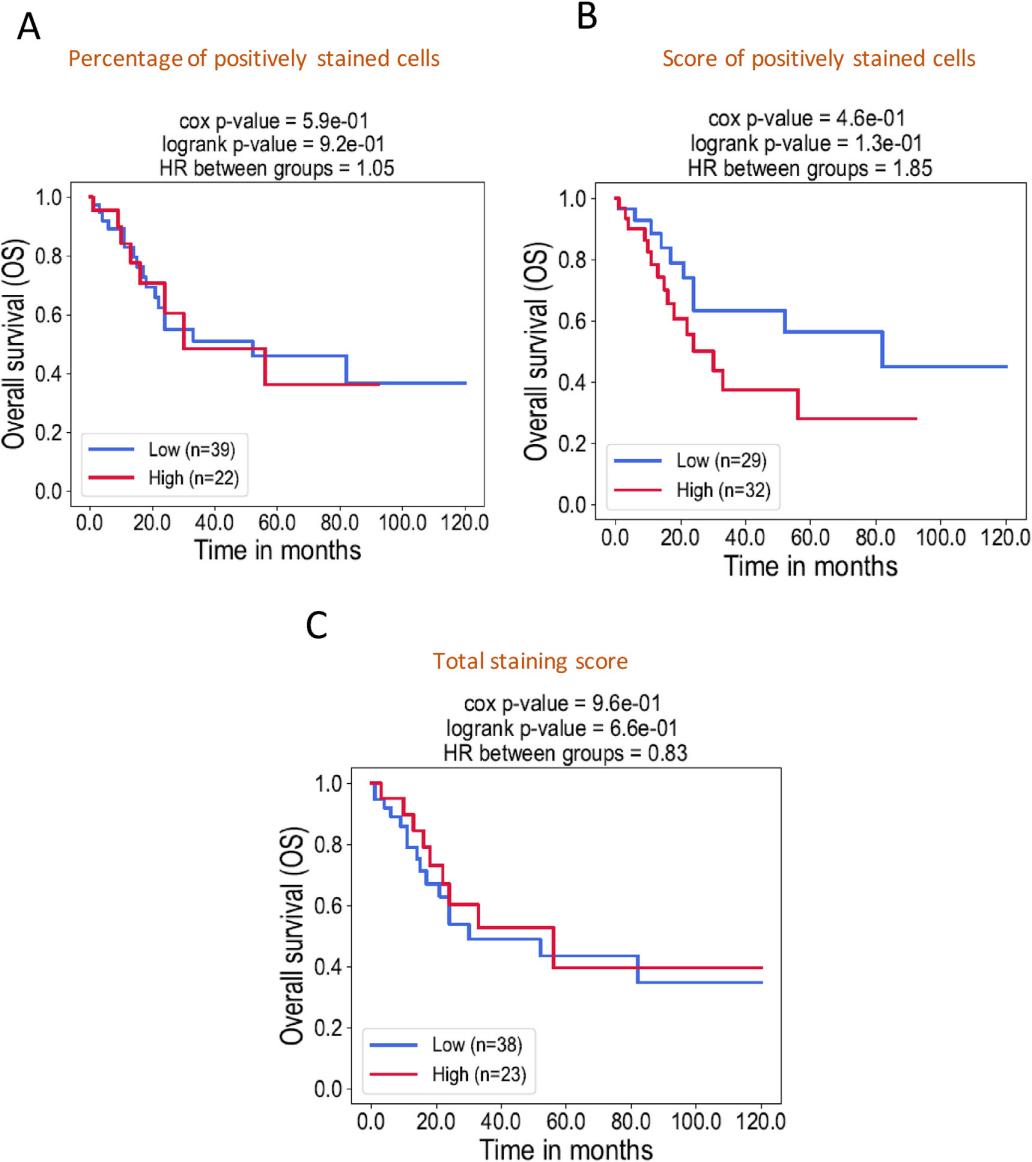
Kaplan-Meier curves for overall survival analysis according to ACAT1 expression in OSCC patients. The stratification of patients according to the percentage of positively stained cells with ACAT1 antibody (A), the score of ACAT1 positively stained cells (B) and the total score of ACAT1 (C) showed no significant association with the survival of OSCC patients. *: p-value <0.05.

We then investigated the association of ACAT1 expression according to the percentage of positively ACAT1 stained cells, the score of percentage and ACAT1’s total staining score with the survival of OSCC patients. Kaplan–Meier analysis revealed no significant difference in overall survival between stratified groups according to high and low levels of ACAT1 (Fig. 4A, B and C).

## 4. Discussion

OSCC is the most common subtype of oral cancer. Overall, cancer is a disease with mitochondrial metabolism alterations which seems to benefit from dietary therapeutic approaches. The association of enzymes responsible for catalyzing ketone bodies with the progression of cancers, including prostate and kidney, has been recently reported [5,8]. Among these enzymes, ACAT1 has been shown as the most interesting and promising candidate and its role with respect to OSCC has yet to be identified. Several studies have shown that cancer cells rely on aerobic glycolysis and that carbohydrate restriction decreases the supply of blood glucose level to cancer cells while enhancing the utilization of fatty acids and ketone bodies in normal cells [12,14,15]. In some cancer patients, the altered expression of certain ketone body metabolic enzymes prevent the tumour cells from obtaining energy from the breakdown of ketone bodies, suggesting that ketogenic diet therapy and carbohydrate restriction could selectively starve and kill cancer cells but not normal cells [12,16]. It is of note that KD therapy in combination with other therapeutic strategies, such as chemotherapy and radiotherapy, could achieve satisfactory results, whereas the positive effects of KD therapy alone are still controversial [16,17]. In the current study, we investigated the ACAT1 protein expression by IHC in a series of 61 OSCC patients and compared the results between OSCC and adjacent pre-tumour tissues. Additionally, the potential of ACAT1 as a prognostic biomarker was evaluated. A decreased ACAT1 expression was observed in high grade and stages of clear cell renal cell carcinoma, and ACAT1 expression was significantly reduced in clear cell renal cell carcinoma compared to adjacent normal kidney tissue [8]. In contrast, an elevated expression of ACAT1 was reported in prostate cancer tissues compared to the adjacent benign tissues, and high levels of ACAT1 expression were observed in high grade and advanced prostate cancer [5]. In our study on OSCC, different expression levels of ACAT1 (low and high expression) were observed among 61 OSCC patients, observed low ACAT1 protein expression in 38 patients (62.3%), suggesting altered mitochondrial metabolism in these OSCC. These variable expression levels of ACAT1 in OSCC tumours could be explained by several hypotheses. First, since ACAT1 is an enzyme located in mitochondria, differences in the number of mitochondria or deficient mitochondria in some OSCC tumours could potentially affect ACAT1 expression. Second, since OSCC tumours are heterogeneous with different metabolic switches, it is possible that tumours with high levels of ACAT1 could generate ATP from both fat and carbohydrates, whereas tumours with low levels of ACAT1 would have to rely mostly on glycolysis and therefore may not respond to KD therapy and carbohydrate restriction. Additionally, the ACAT1 expression in cancerous tissues compared to adjacent pre-tumour tissues did not follow any specific trends in all the patients. This analysis was performed on a subset of 61 patients, which enabled us to stratify them into four groups, including OSCC^low^/Normal^low^, OSCC^low^/Normal^high^, OSCC^high^/Normal^low^ and OSCC^high^/Normal^high^. In 2016, Fan and colleagues reported an upregulated ACAT1 activity despite comparable gene and protein expression in human leukaemia cells, head and neck cancer and lung cancer cells, compared to their corresponding normal cells [6], suggesting that an altered ACAT1 activity could occur without any differences in ACAT1 protein expression (OSCC^low^/Normal^low^ and OSCC^high^/Normal^high^).

Since the expression levels of ACAT1, one of the key enzymes in the ketone body metabolic pathway, are variable between OSCC patients, a high-fat low-carbohydrate diet (ketogenic), which increases the amount of blood ketone bodies, would probably not be able to disrupt the metabolism and therefore would not increase the death of oral squamous cancer cells in all OSCC tumours. These results suggest that ACAT1 staining could help stratify patients into different groups with respect to their potential response to a ketogenic diet, although this statement would still need to be strengthened by analysing other ketolytic enzymes.

In conclusion, our measurements of ACAT1 protein expression did not show any clear common trend regarding the relative expression levels of this enzyme between OSCC and adjacent pre-tumour tissues. Further studies would be required to elaborate on the reason behind these variations of ACAT1 expression among OSCC patients. The main limitation of this study was the evaluation of other ketolytic enzymes including 3-hydroxybutyrate dehydrogenase 1(BDH1), 3-hydroxybutyrate dehydrogenase 2 (BDH2) and succinyl CoA: 3-oxoacid CoA transferase 1 (OXCT1) in the tumour and pre-tumour tissues of the OSCC patients. To conclude on the clinical application of this study, OSCC patients with observed low ACAT1 expression in their tumour compared to the pre-tumour tissue may respond better to ketogenic diet therapy, although further investigations of BDH1, BDH2 and OXCT1 related to the ketolysis would be required.

## Appendix

**Table S1:**

No	Age	Sex	ACAT1	

			OSCC	PT
1	47	F	High	Low
2	56	M	Low	High
3	76	M	High	Low
4	62	M	Low	Low
5	52	M	Low	Low
6	61	M	Low	Low
7	79	F	High	Low
8	83	F	Low	Low
9	53	M	Low	Low
10	33	M	High	High
11	60	M	Low	Low
12	74	M	Low	Low
13	69	F	Low	High
14	57	F	High	High
15	38	F	Low	Low
16	79	F	Low	Low
17	47	F	Low	Low
18	64	F	Low	High
19	54	M	Low	High
20	60	M	High	Low
21	64	F	Low	Low
